# Pretilachlor poisoning: A rare case of a herbicide masquerading as organophosphate toxicity

**DOI:** 10.1002/ccr3.3473

**Published:** 2020-11-03

**Authors:** Olita Shilpakar, Bipin Karki, Bibek Rajbhandari

**Affiliations:** ^1^ Department of General Practice and Emergency Medicine NAMS Bir Hospital Kathmandu Nepal; ^2^ Department of Critical Care Medicine Om Hospital and Research Centre Kathmandu Nepal; ^3^ Department of General Practice and Emergency Medicine Nepal Police Hospital Kathmandu Nepal

**Keywords:** bradycardia, poisoning, vomiting

## Abstract

Acute oral intoxication of pretilachlor, a chloroacetanilide herbicide, in humans can present with similar clinical manifestations of organophosphate toxicity. Clinicians should be aware of such mimickers for proper management of the patient.

## Graphical abstract

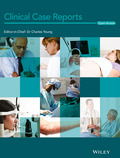

## 1 INTRODUCTION

Pretilachlor is a chloroacetanilide herbicide whose acute intoxication in humans via ingestion has been rarely reported. We report a case of suicidal ingestion of the herbicide with similar clinical manifestations of organophosphate toxicity. Awareness among clinicians regarding such mimickers is the key to proper management of the patient.

Chloroacetanilides are a group of anilide herbicides which commonly include alachlor, butachlor, metachlor, and pretilachlor. Pretilachlor is a broad‐spectrum systemic herbicide with the chemical name 2‐chloro‐2′, 6′‐diethyl‐N‐(2‐propoxyethyl) acetanilide. It has excellent action against annual weeds, sedges, and broadleaf weeds in rice fields.^1^ The mechanism of action of these group of drugs is still not clearly understood but is known to act by inhibiting the biosynthesis of fatty acids, lipids, proteins, flavonoids, etc Chronic exposure to these group of herbicides has shown probable carcinogenic effects; however, acute toxicity from pretilachlor in humans has not been reported yet.^2^ Acute pretilachlor intoxication via ingestion can be mistaken for commonly encountered pesticides like organophosphates resulting in fallacious management of the patient. We report a case of a 42 years old male who presented to the emergency room following suicidal ingestion of the herbicide mimicking clinical manifestations of organophosphate toxicity like vomiting, excessive lacrimation, bowel and bladder incontinence, bradycardia and hypotension, and complete recovery following supportive management.

## 2 CASE REPORT

A 42 years old male presented to the emergency room with the alleged history of suicidal ingestion of an unknown poison 2 hours back following a family dispute. The patient immediately had two episodes of nonbilious nonprojectile vomiting followed by excessive lacrimation, bowel and bladder incontinence, and dizziness within 2 hours of ingestion of the poison. There was no history of shortness of breath, chest pain, loss of consciousness, or seizures. He did not have any past medical or psychiatric history or similar suicidal attempts in the past. On examination in the emergency room, the patient was drowsy but followed verbal commands. He had a radial pulse of 50 beats per minute, blood pressure of 90/50 mm Hg, respiratory rate of 16 breaths/minute, temperature of 98.6 degrees Fahrenheit, and oxygen saturation of 94% in room air. His pupils were three millimeters in size bilaterally and reacting to light. Furthermore, the peculiar garlicky smell of organophosphate was not detected from his breath. His chest auscultation was clear, abdomen was soft and nontender, and the remaining systemic examination did not reveal any abnormality.

With the provisional diagnosis of a possible organophosphorus poisoning, the patient was managed initially by administering an intravenous dose of atropine 0.6 mg following which his heart rate increased to 100 beats per minute showing a normal sinus rhythm in the 12‐lead electrocardiogram. Dermal decontamination was done by removing the patient's vomitus soaked clothing. The skin was cleaned thoroughly with soap water, and gastric lavage was performed simultaneously. A bolus dose of 500 mL of 0.9% normal saline and an intravenous dose of pralidoxime 2 grams over 30 minutes were administered. His investigations comprising a full blood count, arterial blood gas, renal function tests, a random blood sugar level, liver function tests, a coagulation profile, and a serum cholinesterase level were all within normal limits. A chest radiograph and an ultrasonogram of the abdomen and pelvis did not reveal any abnormalities.

A bottle of the herbicide pretilachlor 50% emulsifiable concentrate (EC) was retrieved from his room and was brought to the emergency room by his son after 2 hours. The patient confirmed to have consumed the full bottle containing 250 mL of the herbicide. Close monitoring with continuous supportive management was done for 24 hours with intravenous fluids and antiemetics following which he gradually became asymptomatic. His heart rate remained within the normal range, his blood pressure normalized, and a normal urine output was maintained throughout the hospital stay. Symptoms of lacrimation and incontinence subsided. Repeat blood counts, renal, and liver functions were within normal ranges. He was kept under observation with strict hemodynamic monitoring for the next 2 days which was uneventful. He was discharged from the hospital on the third day after a behavioral counseling session. He was in good health during a 1‐week follow‐up in the outpatient department.

## 3 DISCUSSION

Pretilachlor is a synthetic chloroacetanilide herbicide used effectively in annual grasses and broad‐leaved weeds including *Echinochloa Beauvois*, *Cyperus difformis,* and sedges in rice and paddy fields. Pretilachlor is generally marketed in a 50% emulsifiable concentrate formulation. It appears as a colorless and odorless liquid. It contains an ethoxylated vegetable oil as the emulsifying agent and alkyl aryl sulfonate of calcium salt as the surfactant.^1^

Chronic exposure to chloroacetanilide in vitro and in vivo studies have shown that it has a role in causing neurotoxicity, genotoxicity, and carcinogenicity.^2^, ^3^ Acute oral exposure might have a dissimilar effect on humans, but such cases have been rarely reported to date. A retrospective study of 35 patients with acute oral chloroacetanilide poisoning concluded that although it was found to be of low toxicity in most of the patients, three patients were comatose and one patient died 24 hours after the exposure.^4^ Another study by Lo et al in 113 patients with oral exposure to chloroacetanilides like alachlor and butachlor suggested that around one fourth of the patients were asymptomatic, the rest had vomiting and neurological symptoms ranging from drowsiness to central nervous system depression and three fatalities after manifesting profound hypotension and coma.^5^

Misdiagnosis of pretilachlor poisoning as organophosphorus toxicity in the emergency room could be a major drawback and may lead to faulty management. In our case, the patient displayed several similar clinical features that are encountered with organophosphates like vomiting, excessive lacrimation, bowel bladder incontinence, bradycardia, and hypotension. These hypersecretory effects like salivation, lacrimation, urination, defecation, gastrointestinal motility, emesis, and miosis in case of organophosphate poisoning are due to the overstimulation of muscarinic acetylcholine receptors in the parasympathetic system. Acute muscarinic effects on the heart in the form of bradycardia and hypotension can be life‐threatening.^6^ However, there was no miosis or the presence of the characteristic garlicky odor of organophosphorus toxicity which hinted toward an alternative diagnosis. His serum cholinesterase level was also within normal limit which in case of organophosphates are seen to decrease.^6^

No antidote is yet known to be available for pretilachlor poisoning, so the mainstay of treatment is symptomatic with initial stabilization of the patient, decontamination, intravenous fluid resuscitation, and close hemodynamic monitoring.^7^ In our case, symptomatic bradycardia was alleviated with atropine sulfate administration and the patient became normotensive following intravenous fluids. Therefore, further studies are recommended which would elaborate more on the mechanism of action of this drug on the human body and the definitive management to combat its toxic effects.^8^

## 4 CONCLUSION

This case highlights that despite exhibiting similar clinical manifestations like that of organophosphorus poisoning, initial stabilization, close monitoring, and supportive treatment are the three important aspects for a speedy recovery in pretilachlor poisoning. Education and awareness among the treating physicians regarding mimickers like organophosphates are an important point to be taken into consideration from this case report. The importance of retrieving the poison container wherever possible for the identification of the active compound is emphasized. Redesigning of containers with precautionary warning labels for the general public and restricted availability of this herbicide over the counter are highly recommended.

## CONFLICT OF INTEREST

None declared.

## AUTHOR CONTRIBUTIONS

OS: was involved in the patient care and management. OS and BK: prepared the initial draft of the manuscript. OS, BK, and BR: edited the draft and reviewed the manuscript. All authors approved the final version of the manuscript and agreed to be accountable for any aspects related to the accuracy or integrity of the work.
